# MAP4K signaling pathways in cancer: roles, mechanisms and therapeutic opportunities

**DOI:** 10.1038/s12276-025-01544-8

**Published:** 2025-10-01

**Authors:** Chyntia Sherlyn Bunardi, Minseo Yeom, Prisca Kosasih, Han Han, Wenqi Wang, Gayoung Seo

**Affiliations:** 1 Department of Integrated Biomedical Science, Soonchunhyang Institute of Medi-bio Science (SIMS), Soonchunhyang University, Cheonan, South Korea; 2 Department of Biotechnology, Atma Jaya Catholic University of Indonesia, Jakarta, Indonesia; 3 Department of Pathophysiology, TaiKang Medical School (School of Basic Medical Sciences), TaiKang Center for Life and Medical Sciences, Wuhan University, Wuhan, China; 4 Department of Developmental and Cell Biology, University of California, Irvine, Irvine, CA USA

**Keywords:** Cancer metabolism, Metabolic disorders, Neurological disorders

## Abstract

The MAP4K family, consisting of seven kinases (MAP4K1–7), plays crucial roles in regulating diverse cellular processes, including proliferation, differentiation, migration and apoptosis. Recent studies have highlighted their involvement in multiple signaling pathways such as mitogen-activated protein kinase, Jun N-terminal kinase and Hippo, implicating them in conditions such as cancer, autoimmune and metabolic disorders and neurodegenerative diseases. Notably, MAP4K proteins have demonstrated significant roles in cancer development and progression, including tumor growth, metastasis and immune modulation. Here we summarize current insights into the roles of individual MAP4K members in cancer and other diseases, emphasizing their distinct and overlapping functions within key signaling networks. Furthermore, we discuss the therapeutic potential of targeting MAP4K family members for cancer treatment. These kinases represent promising targets for developing novel therapies for cancer and related diseases. Future research is essential to clarify the specific molecular mechanisms of MAP4K proteins in cancer and to explore their broader relevance in health and disease.

## MAP4K kinases drive cancer progression and therapeutic potential

The MAP4K family of proteins plays a crucial role in various cellular processes, including cell growth and survival. The Review explores how different MAP4K proteins contribute to cancer progression and other diseases. Researchers used various methods, including genetic analysis and experiments on cell lines, to investigate the roles of MAP4K proteins. They found that these proteins are involved in key signaling pathways that regulate cell behavior. For example, MAP4K1 affects immune cell activation, whereas MAP4K4 is linked to cancer cell movement and growth. The Review highlights that MAP4K proteins can act as both promoters and suppressors of cancer, depending on the context. This dual role makes them potential targets for new cancer therapies. The researchers suggest that developing specific inhibitors for these proteins could lead to more effective treatments for cancer and other diseases.

This summary was initially drafted using artificial intelligence, then revised and fact-checked by the author.

## Introduction

MAP4Ks in mammals belong to the serine/threonine kinase Ste20-like family, comprising seven members: MAP4K1 (hematopoietic progenitor kinase 1 (HPK1)), MAP4K2 (germinal center kinase (GCK)), MAP4K3 (GCK-like kinase (GLK)), MAP4K4 (hepatocyte progenitor kinase-like kinase (HGK)), MAP4K5 (KHS) kinase homologous to SPS/STE20, MAP4K6 (MINK1) and MAP4K7 (TRAF2 and NCK-interacting kinase (TNIK))^1^. These kinases act as upstream regulators in the mitogen-activated protein kinase (MAPK) signaling cascade, including the Jun N-terminal kinase (JNK) pathway, and participate in key cellular processes such as proliferation, survival, apoptosis and migration^2^. These kinases can autophosphorylate, leading to either the activation or inhibition of downstream signals^3^.

Based on domain structures, mammalian Ste20-like kinases are classified into two subfamilies: p21-activated kinases and GCKs, with MAP4Ks belonging to the GCK subfamily. Several GCK subgroups can activate MAP3K cascades, ultimately triggering JNK activation^1^. This intricate signaling network, including the MAPK pathway, enables cells to translate extracellular cues into diverse intracellular responses. Among the three principal MAPK pathways, the JNK pathway is particularly involved in regulating cellular activities^4^ and can promote cancer cell growth by interacting with signaling molecules such as nuclear factor kappa B (NF-κB) and JAK/STAT, thereby contributing to cell survival^5^. Structurally, MAP4Ks possess a conserved N-terminal kinase domain and commonly include additional regulatory motifs such as coiled-coil regions and a C-terminal citron homology (CNH) domain. Although the CNH domain is predicted in all MAP4K members, the degree of sequence conservation and functional relevance vary among subgroups^6^.

Within the MAP4K family, MAP4K4, MAP4K6 (MINK1) and MAP4K7 (TNIK) form the evolutionarily conserved GCK-IV subgroup. These kinases are commonly referred to as ‘happyhour-like’ owing to their structural and functional similarity to *Drosophila happyhour*, a kinase known to modulate EGFR and stress-related signaling pathways^7^. Their CNH domains are well conserved and have been implicated in interactions with small GTPases such as RAP2, contributing to Hippo pathway activation and mechanosensitive signaling^8,9^. By contrast, although MAP4K1–3 and MAP4K5 also contain CNH-like sequences, their specific roles remain poorly defined. Notably, functional studies have demonstrated that MAP4K1–MAP4K3 and MAP4K5 can phosphorylate and activate LATS1/2 kinases similarly to MST1/2, thereby functioning as ‘alternative Hpo-like’ kinases within the Hippo signaling context^10,11^.

Emerging evidence suggests that several MAP4Ks interact with the STRIPAK complex, a multiprotein phosphatase scaffold assembly implicated in Hippo pathway regulation. Proteomics-based proximity labeling has identified interactions between all MAP4K members and STRN4, a core STRIPAK component^12^. However, the functional significance of these interactions has been most extensively characterized for MAP4K4. For example, MAP4K4 modulates Hippo pathway activation and cytoskeletal organization through STRIPAK-dependent associations with PP2A, PKCθ and actin regulators such as VASP and ERM proteins^13,14^. Despite these findings, most experimental evidence focuses on MAP4K4, leaving it unclear whether similar STRIPAK-mediated mechanisms operate across other MAP4K family members. Therefore, caution should be exercised when generalizing STRIPAK-based regulation without further comparative investigation.

In summary, structural and functional diversities exist among MAP4K family members, particularly regarding CNH domain conservation and their specific roles in Hippo signaling and cytoskeletal regulation. Future research is needed to investigate novel mechanisms by which these kinases influence cellular signaling. MAP4Ks may interact with additional signaling molecules or pathways, affecting broader cellular outcomes beyond current understanding. Exploring these mechanisms may reveal new regulatory dimensions in cell growth and differentiation, enhance our knowledge of tissue homeostasis and identify potential therapeutic targets for diseases such as cancer. A deeper understanding of MAP4K signaling will enable better assessment of their mediator roles in cancer progression.

## Main text

### MAP4K family in cancer

**Fig. 1:**
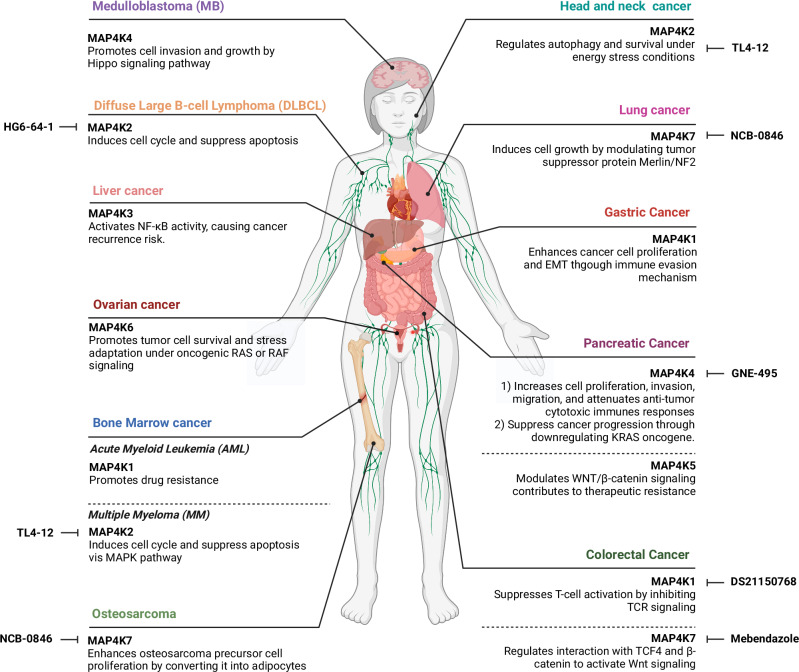
Diverse roles of MAP4K family kinases in cancer progression. This figure illustrates the multifaceted roles of MAP4K family kinases across various cancer types. The key functions include promoting cancer cell proliferation, migration, invasion, drug resistance and immune evasion, as well as regulating apoptosis, autophagy and major signaling pathways such as MAPK, NF-κB, WNT/β-catenin and Hippo. These kinases are also implicated in cancer recurrence, therapeutic resistance and cellular stress adaptation, highlighting their potential as critical therapeutic targets in oncology. Specific inhibitors and targeted therapeutic interventions within MAP4K-associated pathways are also indicated. The figure was created using bioRender.com.

The MAP4K family has emerged as a critical regulator in cancer, playing diverse roles in tumorigenesis, metastasis and immune modulation across various cancer types. This section explores the distinct and overlapping functions of individual MAP4K members in cancer progression and their potential as therapeutic targets^5^ (Fig. 1).

MAP4K1 is known to play an important role in modulating T cell activation, functioning as a negative regulator of T cell receptor (TCR) signaling^15,16^. The inhibition of MAP4K1 enhances T cell activation and improves immune responses against tumors^17^. In addition, MAP4K1 contributes to tumor suppression by promoting T cell proliferation upon stimulation with anti-CD3 and anti-CD28 antibodies. This leads to an increased production of proinflammatory cytokines, thereby amplifying T cell activation and inhibiting tumor growth. To support this strategy, a compound named GNE1858 has been developed, which binds to the ATP-binding pocket of MAPK1. GNE1858 serves as a lead structure for the development of next-generation inhibitors. A study by Wu et al. demonstrated that GNE1858 effectively targets the MAP4K1 kinase domain in a dose-dependent manner, as well as the intrinsic kinase activity of the MAP4K1-SA mutant variant (with a key serine residue substituted with alanine to prevent phosphorylation)^18^.

Another study demonstrated that combining MAP4K1 inhibition with programmed cell-death ligand 1 (PD-L1) blockade can enhance T cell responses against tumor cells with low antigenicity^19^. MAP4K1 dampens the TCR signaling pathway by inactivating the Src homology 2 domain-containing leukocyte protein of 76 kDa (SLP76)^20,21^. This suppression reduces the activation of key downstream pathways, including ERK, NF-κB and c-Jun, all of which are critical for robust T cell responses. The compound DS21150768 has been shown to inhibit MAP4K1, thereby enhancing the activation of these downstream pathways, particularly in response to low-affinity antigens. In vitro studies using OT-1 splenocytes, a model for TCR-mediated immune responses, have demonstrated that DS21150768 significantly boosts T cell activation and cytokine production, specifically IL-2 and IFN-γ. This enhancement is particularly notable under conditions of low-affinity antigen stimulation, which typically elicits weak T cell responses. These findings suggest that DS21150768 can potentiate T cell activity even under suboptimal antigenic conditions. The antitumor effects of DS21150768 were further evaluated in vivo using a mouse model implanted with MC38 tumor cells engineered to express ovalbumin peptides of varying affinities. Tumors expressing low-affinity ovalbumin peptides were generally resistant to immune checkpoint inhibitors such as anti-PD-L1, which had minimal impact on tumor growth when administered alone. Similarly, DS21150768 monotherapy showed limited efficacy. However, combining DS21150768 with anti-PD-L1 significantly suppressed tumor growth, indicating a synergistic effect that overcomes immune resistance in poorly immunogenic tumors. This combination therapy highlights a promising strategy to target cancers that evade immune surveillance due to low tumor antigenicity, thereby broadening the potential applications of immunotherapy in resistant tumor types^19–22^.

Acute myeloid leukemia (AML) is a rapidly progressing myeloid neoplasm characterized by the clonal expansion of primitive hematopoietic stem cells in the bone marrow. Recent studies have shown that the overexpression of *MAP4K1* is associated with poor prognosis in AML, as it enhances drug resistance by regulating the MAPK pathway through Jun and JNK signaling factors^23,24^. One study conducted RNA sequencing profiling on AML cell lines with varying responses to homoharringtonine (HHT) treatment—specifically, HHT-resistant and HHT-sensitive lines. HHT is a plant-derived alkaloid and protein synthesis inhibitor approved for treating chronic and AML; it functions by preventing elongation during translation^25^. A bioinformatic analysis of RNA sequencing data was used to identify gene expression differences linked to MAP4K1’s role in drug resistance. The study found that the knockdown of MAP4K1 increased the sensitivity of AML cells to HHT treatment by inhibiting JNK and Jun activity. This, in turn, led to upregulation of the cell cycle regulators p21 and p27, thereby modulating AML progression. The downregulation of MAP4K1 was shown to induce G0/G1 phase cell cycle arrest, driven by the increased expression of p21 and p27. Supporting these findings, sequencing data revealed that MAP4K1 and c-Jun were significantly upregulated in HHT-resistant cell lines, highlighting their critical role in mediating resistance to HHT^23^.

Another study by Wu et al. demonstrated that MAP4K1 downregulation can inhibit the progression of gastric cancer by suppressing cell proliferation, migration and epithelial–mesenchymal transition (EMT)^26^. The long noncoding RNA (IncRNA) *DLX6-AS1* known to be dysregulated at different levels in several cancers, including gastric, non-small-cell lung and bladder cancers, plays a significant regulatory role^27^. In gastric cancer, *DLX6-AS1* modulates FUS expression, which in turn enhances *MAP4K1* mRNA stability through interaction with the FUS protein. This stabilization maintains MAP4K1 protein levels and promotes oncogenic behaviors, including cell migration and EMT^26^.

Programmed cell-death protein 4 (PDCD4) functions as a tumor suppressor by inhibiting the c-Jun pathway, which is associated with cancer cell proliferation and invasion^28^. USP4 directly interacts with PDCD4, removing ubiquitin chains that target PDCD4 for proteasomal degradation. The stabilization of PDCD4 results in the reduced expression of key genes involved in tumor cell proliferation and invasion^29^. Recent studies have shown that the overexpression of PDCD4 in RKO colon carcinoma cells suppresses MAP4K1 transcription, leading to decreased JNK activity and reduced c-Jun phosphorylation. PDCD4 particularly inhibits the MAP4K1 promoter without affecting other pathways, such as ERK or NF-κB, thereby reducing tumor cell invasion^30,31^.

MAP4K2 plays a distinct role in mediating YAP-independent autophagy and promoting cell survival in response to energy stress^32^. Particularly, MAP4K2 autophosphorylation at serine 170 can be triggered by glucose starvation, facilitating autophagosome–lysosome fusion during autophagic flux, which contributes to head and neck cancer (HNC) progression. An orthotopic xenograft model, in which CAL27 cells were implanted into the tongues of mice, was used to investigate the impact of MAP4K2 on HNC progression. The inhibition of MAP4K2 led to a significant reduction in tumor growth, underscoring its critical role in tumorigenesis. Moreover, patient-derived xenograft models were used to evaluate the therapeutic potential of TL4-12, a MAP4K2 inhibitor. Treatment with TL4-12 significantly reduced tumor size and weight in these models, highlighting the promise of MAP4K2 inhibitors as potential therapeutic agents for HNC^32^.

Diffuse large B cell lymphoma (DLBCL) is the most common subtype of non-Hodgkin lymphoma, characterized by rapidly growing tumors originating from mature B cells^33^. The inhibition of MAP4K2 has emerged as a promising therapeutic approach in DLBCL, as it induces cell cycle arrest and apoptosis. HG6-64-1, a chemical inhibitor of MAP4K2, significantly suppresses tumor growth and prolongs survival in mouse models^34^. Clinically, high MAP4K2 expression correlates with poor outcomes, whereas patients lacking MAP4K2 expression exhibit longer progression-free survival. Li et al. demonstrated that MAP4K2 plays a critical role in the survival and proliferation of RAS-mutant multiple myeloma (MM) cells, a malignancy of terminally differentiated plasma cells in the bone marrow that commonly leads to anemia, bone destruction and kidney dysfunction^35,36^. The knockdown of MAP4K2 significantly inhibited MM cell growth and induced apoptosis both in vitro and in vivo. Mechanistically, MAP4K2 regulates the MAPK signaling pathway by activating MKK4/7 and JNK, which are essential for MM cell survival. The inhibition of MAP4K2 also reduced the expression of key transcription factors, including IKZF1/3, c-MYC and BCL-6, all of which are crucial for MM progression. Preclinical studies have shown that MAP4K2 inhibitors, such as TL4-12, can effectively suppress tumor growth and induce apoptosis in RAS-mutant MM cells. Moreover, MAP4K2 inhibition overcomes resistance to immunomodulatory drugs such as lenalidomide, offering a promising treatment strategy for refractory MM^35^.

In addition to its broad expression profile, MAP4K3 is involved in several essential cellular functions, including mTORC1 signal transduction, cell proliferation, apoptosis, autophagy and immune regulation^37–39^. The overexpression of MAP4K3 has been associated with cancer recurrence and poor prognosis in various malignancies^39–41^. Supporting this, a study by Ho et al. investigated the tumorigenic effects of MAP4K3 in hepatocellular carcinoma (HCC) and demonstrated that increased MAP4K3 expression enhances IκB kinase (IKK) phosphorylation and promotes nuclear translocation of p65, thereby activating NF-κB signaling^42^. Notably, malignant tissues from patients with HCC exhibited higher levels of phosphorylated IKK compared with adjacent noncancerous tissues^43^.

The role of MAP4K3 in cancer development was further explored by Chuang et al., who found that MAP4K3 promotes tumor metastasis and cell migration via interaction with a novel scaffold protein, IQ-motif-containing GTPase-activating protein 1 (IQGAP1). MAP4K3 directly binds to and phosphorylates IQGAP1 at Ser480 residue, which facilitates the activation of the Rho family GTPase Cdc42. This activation occurs through the interaction of MAP4K3 with IQGAP1 at the leading edge of migrating cells, resulting in enhanced cell migration and metastasis. Furthermore, transgenic overexpression of MAP4K3 significantly promoted distant metastasis in a lung cancer mouse model, whereas the absence of IQGAP1 completely abolished MAP4K3-induced lung metastasis^41^. These findings suggest that the MAP4K3–IQGAP1 axis could serve as a promising therapeutic target in cancer treatment.

MAP4K4 has emerged as a prominent target in therapeutic intervention studies due to its involvement in various cellular processes, including cell proliferation, survival, inflammation, stress responses, cytoskeletal dynamics and ion transport^44–47^. Supporting its role in cell proliferation, primary mouse lung endothelial cells lacking MAP4K4 exhibited enhanced proliferation, accompanied by alterations in RAS-mediated G2/M transition signaling^46^. Interestingly, a study by Collins et al. found that MAP4K4 knockdown successfully inhibited the migration of multiple carcinoma cell lines, highlighting its role in cell motility and the therapeutic potential of targeting the MAPK pathway in cancer progression. Further research has expanded the understanding of MAP4K4’s role in stress responses and inflammation. Notably, MAP4K4 was shown to activate TNF-α expression through a proinflammatory pathway that operates independently of JNK1/2, p38 and NF-κB signaling. This identifies MAP4K4 as a novel therapeutic target for suppressing TNF-α expression, particularly in lipopolysaccharide-induced macrophage inflammatory responses. Definitive evidence of MAP4K4’s pivotal role came from siRNA-mediated silencing experiments in macrophages, which conferred protection against lipopolysaccharide-induced lethality in mice by significantly reducing the production of TNF-α and interleukin-1β^44^.

Aligned with its critical roles in numerous cellular processes, MAP4K4 has been implicated in the development of nonmalignant and malignant diseases, including metabolic disorders, cardiovascular diseases, inflammation and cancer^13,44–51^. The dysregulation of MAP4K4 function has been associated with cancer initiation and progression, as it can activate proproliferative pathways, suppress antitumor cytotoxic immune responses and promote cell invasion and migration by altering cytoskeletal structure, actin dynamics and membrane protrusion formation. A growing body of evidence has revealed that MAP4K4 coordinates diverse signaling pathways governing inflammation, insulin sensitivity, lipogenesis, cytoskeletal remodeling and autophagy. These functions have been elucidated through studies in various biological contexts, including adipose tissue, endothelial cells, immune cells and multiple cancer models. For instance, MAP4K4 modulates cytoskeletal dynamics by regulating ERM–integrin interactions and actin-rich protrusion^13,45,47^. It contributes to insulin resistance through inflammatory cascades^44,48,49^ and suppresses adipogenic differentiation via nutrient-sensitive signaling pathways such as AMPK–mTOR^50^. In addition, MAP4K4 plays an essential role in autophagy regulation under metabolic and oncogenic stress^51^.

Furthermore, MAP4K4 plays a significant role in cancer metastasis, particularly through its involvement in the regulation of collective cell migration^52–54^. Recent studies using A431 carcinoma cells demonstrated that MAP4K4 facilitates collective migration by modulating mechanical forces at cell–cell and cell–matrix adhesion sites. The inhibition of MAP4K4, particularly via the small-molecule inhibitor GNE-495, markedly reduces migration speed, stabilizes focal adhesions and alters cytoskeletal dynamics, ultimately impairing the coordination of migrating cell clusters^52^. In addition, MAP4K4 phosphorylates MLK3, a process that promotes pancreatic cancer cell proliferation, migration and colony formation. Treatment with GNE-495 reduces tumor burden and prolongs survival in a pancreatic cancer mouse model^54^. These findings underscore the importance of MAP4K4’s kinase activity in reducing tension at adhesion sites to support collective migration. Conversely, MAP4K4 overexpression can disrupt cluster cohesion and promote cell scattering, potentially contributing to metastatic spread^52,54^.

The interaction between MAP4K4 and the STRIPAK complex, particularly with STRN3, drives medulloblastoma (MB) progression^45^. MAP4K4 and STRN3 cooperate to promote tissue invasion and regulate tumor growth by modulating Hippo signaling. STRN3 facilitates the coupling of MAP4K4 to the phosphatase PP2A, thereby suppressing MAP4K4’s tumor-suppressive activity and enhancing the proinvasive behavior of MB cells. This interaction enables FGFR-driven MB cell motility and invasion and regulates cytoskeletal dynamics through the phosphorylation of key proteins such as VASP and PKCθ. These findings suggest that targeting the MAP4K4–STRN3 axis represents a promising therapeutic strategy for MB^45^. Moreover, Kim et al. investigated the interaction between MAP4K4 and SRIPAK, confirming that STRN4, a member of the STRIPAK complex, associates with the SV40 small T antigen (ST). STRN4 was shown to be essential for ST-PP2A-induced cell transformation via the activation of the Hippo pathway effector YAP^14^.

MAP4K4 overexpression has been reported in various cancer types, including colorectal, gastric, pancreatic, lung, ovarian epithelial cancers and HCC^3,55,56^. However, a recent study by Juin et al. revealed a dichotomous role for MAP4K4 in pancreatic ductal adenocarcinoma (PDAC), an aggressive and lethal cancer with limited early detection and poor prognosis^57^. The loss of MAP4K4 accelerated PDAC onset and progression by inducing the hyperactivation of ERK and AKT signaling, which are critical downstream effectors of the oncogene KRAS^58^. Although MAP4K4 deficiency led to faster tumor development, it simultaneously reduced metastatic potential by impairing cell invasion, matrix remodeling and metastatic seeding. Although MAP4K4 remains a compelling target for controlling PDAC metastasis, its inhibition may inadvertently enhance primary tumorigenesis, highlighting the need for careful evaluation in therapeutic applications^56^.

Decreased MAP4K5 expression has been significantly associated with reduced overall survival in PDAC. This reduction is strongly correlated with dysregulation of the Wnt–β-catenin signaling pathway, which is crucial for maintaining epithelial cell identity and cell–cell junction integrity. The disruption of this pathway can trigger EMT, a key process that contributes to therapeutic resistance in pancreatic cancer. The knockdown of MAP4K5 in pancreatic cancer cell lines leads to decreased CDH1 mRNA expression, highlighting the essential role of MAP4K5 in regulating E-cadherin expression and EMT^59^.

MAP4K6 has been implicated in mediating tumor gene-induced growth arrest in ovarian epithelial cells via reactive oxygen species levels and p38 MAPK activation. Nicke et al. reported that MAP4K6 facilitates RAS-induced growth inhibition in human ovarian surface epithelial cells following oncogenic RAS and RAF activation. This leads to activation of p38 MAPK, increased levels of the cyclin-dependent kinase inhibitor p21^WAF1/CIP1^ and reduced cyclin A levels, thereby inducing cell cycle arrest. Furthermore, the activation of p38 MAPK pathway is mediated through MAP3K5 (Ask1), which activates MKK3 and MKK6, ultimately resulting in p38 MAPK phosphorylation. These results suggest that MAP4K6 functions as a tumor suppressor, contributing to protective mechanisms against malignancy^60^.

MAP4K7 has also been identified as a critical activator of the Wnt signaling pathway^61–63^. A small-molecule MAP4K7 inhibitor, NCB-0846, has demonstrated anticancer effects against metastatic colorectal cancer^61,63^. This compound directly binds to TCF4 and β-catenin through the kinase and intermediate domains of MAP4K7. During this interaction, MAP4K7 specifically phosphorylates the conserved serine 154 residue of TCF4 (ref. ^63^). Moreover, a separate study showed that inhibiting MAP4K7 kinase activity using small-molecule inhibitors is a promising therapeutic strategy in colorectal cancer, as it disrupts the interaction of MAP4K7 with TCF4 and β-catenin^62^. Loss-of-function mutations in the Wnt signaling inhibitor adenomatous polyposis coli, along with activating mutations in β-catenin, are recognized as major drivers of colorectal cancer development. In this context, Tan et al. investigated a novel mechanism of action for the FDA-approved anthelmintic drug mebendazole, which targets MAP4K7 within the Wnt signaling cascade associated with colorectal cancer^63^. The study concluded that mebendazole exhibits high-affinity binding to MAP4K7 and selectively inhibits its kinase activity.

MAP4K7 is amplified in ~50% of lung squamous cell carcinoma (LSCC) cases, a histologic subtype of non-small-cell lung cancer commonly associated with tobacco use and often lacking effective targeted therapies^64,65^. The genetic depletion or pharmacological inhibition of MAP4K7 significantly reduces the growth of lung squamous carcinoma cells in vitro and in vivo, highlighting MAP4K7’s oncogenic role. This effect is mediated through the regulation of the tumor suppressor protein Merlin (also known as NF2). MAP4K7 directly phosphorylates Merlin at serine residues S13 and S15, which subsequently activates focal adhesion kinase (FAK), a key downstream effector. FAK is a nonreceptor tyrosine kinase that translocates between the cytoplasm and nucleus and participates in several signaling pathways that drive tumorigenesis. In addition, phosphopeptide mapping and in vitro kinase assays have revealed that MAP4K7 phosphorylates Merlin at additional sites, including T272 and T576. The knockdown of Merlin in LSCC cells leads to reduced FAK activation, and the dual knockdown of MAP4K7 and Merlin further decreases FAK activity. Following MAP4K7 knockdown, YAP levels are also diminished in LK2 and NCI-H520 cells, consistent with Merlin’s known role in negatively regulating the pro-oncogenic transcription factor YAP. Moreover, the siRNA-mediated knockdown of Merlin, FAK and YAP significantly reduces cell viability in LK2 and NCI-H520 cells. These findings underscore the critical role of Merlin phosphorylation in sustaining FAK activation and stabilizing YAP, offering a mechanistic explanation for how MAP4K7 supports LSCC cell survival^64^.

The inhibition of MAP4K7 using small-molecule inhibitors such as NCB-0846 has been shown to suppress the growth of LSCC, colorectal cancer and leukemia stem cells. This approach represents a promising targeted therapy, particularly for LSCC, which currently lacks approved targeted treatments and still relies on conventional therapies such as chemotherapy and radiotherapy^66^. The treatment of LSCC with NCB-0846 results in a substantial reduction in cell viability, including in patient-derived xenografts from established LSCC cases. The reduction in viability is largely attributed to the catalytic inhibition of MAP4K7. Notably, this effect can be reversed by expressing an inhibitor-resistant mutant form of MAP4K7 (MAP4K7^V31W^). In addition, NCB-0846 has minimal impact on cells with near-complete MAP4K7 knockdown, further confirming the specificity of its action^64^.

Osteosarcoma, a rare malignant bone tumor primarily affecting adolescents and young adults, is characterized by aggressive local invasion and a high risk of metastasis. The disease is also associated with impaired differentiation and the formation of osteosarcoma precursor cells^67,68^, which are essential for the transcriptional activation of MAP4K7 and Wnt signaling target genes—factors that may contribute to the poor prognosis. RNA interference or pharmacological inhibition of MAP4K7 has been shown to suppress osteosarcoma cell proliferation by inducing their differentiation into adipocytes, both in vitro and in vivo. This lineage conversion is associated with decreased expression of key transcription factors that maintain the embryonic stem cell phenotype (SOX2, NANOG, OCT4 and MYC)^69^, along with increased expression of PPARγ, a master regulator of adipogenesis^70^. Hirozane et al. identified MAP4K7 as a molecular target in osteosarcoma and demonstrated that its silencing or inhibition via NCB-0846 effectively reduces MAP4K7 expression and inhibits the proliferation of various osteosarcoma cell lines, including U2OS, NOS-10, MNNG/HOS, NOS-1, HsOS, HuO9N2 and NY^68^. These findings suggest that MAP4K7 is essential for maintaining osteosarcoma cell proliferation and stemness, highlighting its potential as a therapeutic target for osteosarcoma^69^.

### MAP4K family in other diseases, including autoimmune disorders, neurodegenerative diseases, viral infections and diabetes

Beyond their established roles in cancer, MAP4K family members also contribute to the pathogenesis of several nonmalignant diseases, including autoimmune disorders, neurodegenerative diseases, viral infections and metabolic disorders such as diabetes. This section explores their involvement in these conditions, with a focus on underlying mechanisms and potential therapeutic relevance (Table 1).

**Table 1:** Disease associations and therapeutic opportunities related to MAP4K family kinases.

MAP4K family members	Associated diseases	Mechanisms	Potential therapeutics (inhibitors, small molecules)
MAP4K1 (HPK1)	Cancer: AML, gastric cancer and colorectal cancer	T cell activation inhibition and MAPK pathway regulation	GNE1858 and DS21150768
MAP4K2 (GCK)	Cancer: HNC and DLBCL	Autophagy activation and MAPK pathway regulation	TL4-12 and HG6-64-1
MAP4K3 (GLK)	Cancer: HCC	Activation of NF-κB and mTORC1 pathway	–
	Autoimmune diseases: SLE	PKCθ activation via TCR signaling	–
MAP4K4 (HGK)	Cancer: pancreatic cancer, colorectal cancer and MB (Note: dichotomous role in pancreatic cancer)	Hippo pathway modulation and cell migration regulation	GNE-495
	Neurodegenerative disorders: amyotrophic lateral sclerosis and motor neuron degeneration	JNK inhibition, neuroprotection	MAP4K4 inhibitor 29 (MAP4K4i)
MAP4K5 (kinase homologous to SPS1/STE20 (KHS1))	Cancer: pancreatic cancer	Dysregulation of WNT/β-catenin pathway	–
	Viral infections: SARS-CoV-2	Interaction with 3CL^pro^ protease	–
	Metabolic diseases: type 2 diabetes	JNK pathway activation, regulation of insulin resistance	–
MAP4K6 (*misshapen*-like kinase (MINK)) *Misshapen*/Nck-related kinase	Cancer: ovarian cancer (Note: tumor-suppressor role)	Ras-mediated MAPK pathway	–
	Neurodegenerative disorders: Alzheimer’s disease	Regulation of DLK/JNK signaling, neuroprotection	–
MAP4K7 (TNIK)	Cancer: lung cancer, colorectal cancer and osteosarcoma	Activation of WNT signaling pathway	NCB-0846
	Metabolic diseases: obesity and lipid metabolism disorders	Regulation of glucose and lipid metabolism	–

MAP4K1 regulates T cell activation and MAPK signaling in cancers such as AML and colorectal cancer, with inhibitors such as GNE1858 identified. MAP4K2 is associated with cancers and autoimmune diseases, modulating autophagy and MAPK signaling. MAP4K3, implicated in cancer, neurodegeneration and diabetes, influences the NF-κB, mTORC1 and Hippo pathways, with GNE-495 proposed as a potential therapeutic agent. MAP4K4 contributes to cancer progression, metabolic disorders and viral infections via WNT and JNK signaling. MAP4K5 functions as a tumor suppressor in ovarian cancer, whereas MAP4K6 exhibits neuroprotective effects in Alzheimer’s disease. MAP4K7 enhances WNT signaling in cancers, with NCB-0846 under investigation as a treatment option.

MAP4K3 is broadly expressed in various tissues, including the heart, brain, placenta, lung, liver, skeletal muscle, kidney and pancreas^71^. Similar to other MAP4K proteins, MAP4K3 overexpression has been associated with the development of multiple autoimmune disorders^39^. This association is attributed to the hyperactivation of PKCθ, which subsequently activates the transcription factor NF-κB via TCR signaling^72^. Proper regulation of this signaling pathway is crucial, as NF-κB plays a central role in survival, activation and differentiation of innate immune cells and inflammatory T cells^72^. Moreover, PKCθ is essential for mounting effective host defense responses and initiating inflammation^73^. The dysregulation of this pathway may contribute to the onset of autoimmune disorders and cancer^74^, positioning MAP4K3 as a key modulator of immune homeostasis and autoimmunity.

As a pivotal regulator of immune function, MAP4K3 overexpression in T cells promotes IL-17A production and excessive T cell activation, driving autoimmune inflammatory disorders such as systemic lupus erythematosus (SLE), a chronic autoimmune condition affecting multiple organ systems, including the skin, kidneys and central nervous system^75^. Supporting this, genomic sequencing studies by Chuang et al. demonstrated that several GLK germline and somatic variants lead to GLK overexpression by enhancing mRNA or protein stability in patients with SLE^76,77^.

MAP4K4 is also implicated in neurobiological processes and neurodegenerative diseases^78^. Its inhibition is strongly associated with enhanced motor neuron survival as MAP4K4 contributes to motor neuron degeneration by triggering ER-stress-mediated neurodegeneration. MAP4K4 regulates motor neuron degeneration by inducing ER-stress-mediated neurodegeneration, indicating that MAP4K4 inhibition is strongly correlated with motor neuron survival^79^. Inhibiting MAP4K4 not only promotes motor neuron survival by suppressing JNK3-c-Jun-driven apoptosis but also preserves neurite integrity and reduces mutant SOD1 levels via autophagy activation. These findings underscore the therapeutic promise of MAP4K4 inhibitors, particularly MAP4K4 inhibitor 29 (MAP4K4i), which improves the survival of motor neurons derived from embryonic stem cells and induced pluripotent stem cells^51^.

In addition, Chen et al. demonstrated that MAP4K4 plays a crucial role in microcirculatory dysfunction associated with diabetic cardiomyopathy, a cardiac complication of diabetes mellitus characterized by myocardial structural and functional abnormalities independent of hypertension or coronary artery disease^80^. Under diabetic conditions, MAP4K4 upregulation significantly impairs mitochondrial function and morphology and promotes endothelial ferroptosis by activating SNO-Drp1 through suppression of GPX4 expression, ultimately causing microvascular injury. These findings position MAP4K4 as a key regulator of SNO-Drp1 and suggest it as a promising therapeutic target for treating diabetic cardiomyopathy in diabetes^48^.

MAP4K5 has been reported as a contributor to viral infections. It contains ten Leu–Gln motifs, with at least three optimal sequences recognized by the 3-chymotrypsin-like main protease (3CL^pro^), a critical enzyme required for severe acute respiratory syndrome coronavirus 2 (SARS-CoV-2) replication. 3CL^pro^ disrupts MAP4K5 kinase activity by cleaving and separating its Ser/Thr kinase domain from the CNH domain. Consequently, 3CL^pro^ also disrupts the transcriptional components of the Hippo pathway, including YAP1 (ref. ^81,82^). Particularly, the cleavage of YAP1 at Gln133 generates a C-terminal fragment resembling the transcriptionally inactive YAP1 isoform 4, which suppresses IRF3 translocation and impairs innate antiviral responses. The repeated inactivation of YAP1 via loss of its S127 phosphorylation/14-3-3ε binding site, together with MAP4K5 inactivation (as an upstream regulator) and targeting of downstream transcription factors CREB1 and ATF1, highlights the strategic inhibition of Hippo-regulated gene transcription and TANK-binding kinase 1 (TBK1) activity as essential mechanisms facilitating SARS-CoV-2 infection^82^.

MAP4K5 is also implicated in the pathogenesis of diabetes. Its link to type 2 diabetes mellitus involves the TNF-α-induced JNK signaling pathway. As a key mediator of insulin resistance, TNF-α activates MAP4K5, which then interacts with TRAF2 to initiate downstream signal transduction^83^. A study by Gu et al., involving 1399 individuals from the Chinese Han population, found that the MAP4K5-882 AA genotype was associated with a lower risk of type 2 diabetes mellitus, particularly in individuals with larger waist circumferences^84^.

Beyond metabolic disorders, recent studies suggest that MAP4K signaling also contributes to the metabolic plasticity of cancer cells. For example, MAP4K4 and MAP4K5 have been implicated in regulating lipogenesis and anabolic processes in tumor cells. MAP4K4 suppresses adipogenic lipogenesis via AMPK- and mTOR-dependent inhibition of SREBP-1, thereby maintaining tumor cells in a proliferative state^85^. The inhibition or loss of MAP4K4 enhances adipocyte differentiation and lipid accumulation, a phenomenon also observed in certain sarcomas and leukemias^13^. These findings highlight the dual role of MAP4Ks in promoting oncogenic signaling while suppressing terminal differentiation. Thus, the pharmacological inhibition of MAP4Ks may create a therapeutic vulnerability by pushing malignant cells toward metabolically unfavorable or differentiated states, representing a potential strategy to disrupt tumor progression.

Broce et al. have also demonstrated a strong association between cardiovascular disease and the development of Alzheimer’s disease^86,87^. MAP4K6 is implicated in cardiovascular risk factors and Alzheimer’s pathology. According to an ‘AD-by-proxy’ cohort analysis, genetic variants associated with MAP4K6 are linked to an increased risk of Alzheimer’s disease^88^.

MAP4K6 and MAP4K4 have been identified as upstream regulators of the dual leucine zipper kinase (DLK)–JNK signaling pathway in neurons^89^. MAP4K6, previously identified as a component of the postsynaptic density, plays a role in regulating synaptic function, and DLK is a key mediator of stress-induced JNK signaling in neurons^90^. MAP4K6 modulates the DLK/JNK pathway by directly activating MAP3 kinases such as DLK through phosphorylation of their activation loop. This mechanism was explored by Larhammar et al. using selective MAP4K4 inhibitors (GNE-495, MAP4Ki_10, MAP4Ki_29 and MAP4Ki_26) in combination with loss-of-function approaches^89^. Their study used a high-content imaging screen based on a nerve growth factor (NGF) withdrawal model, which mimics the competitive environment faced by TrkA-expressing dorsal root ganglion neurons during development. The results revealed that NGF deprivation increased the phosphorylation of c-Jun via DLK–JNK pathway activation. Supporting these findings, treatment with MAP4K4 inhibitors (GNE-495, MAP4Ki_29, MAP4Ki_10 and MAP4Ki_26) and the DLK inhibitor GNE-3511 significantly reduced DLK/JNK pathway activation following NGF withdrawal. These results suggest that inhibiting MAP4K6 can block stress-induced retrograde JNK signaling and confer neurodegeneration, highlighting MAP4K6 as a promising therapeutic target in neurodegenerative diseases^89^.

MAP4K7 has been shown to be regulated by adenosine 5′-monophosphate-activated proteins in skeletal muscle in response to human physical activity. It is also modulated by several signaling pathways, including JNK, NF-κB, Wnt and AMPK. Pham et al. identified MAP4K7 as a key regulator of glucose and lipid metabolism, demonstrating that genetic ablation of MAP4K7 in mice or flies significantly alters cellular metabolism. This alteration results in reduced activation of lipogenic programs typically triggered by high-fat, high-sucrose or high-sugar diets. Notably, MAP4K7-knockout mice exhibit increased ambulatory activity and are protected against diet-induced obesity, peripheral insulin resistance and hepatic lipid accumulation. Furthermore, MAP4K7 loss-of-function variants are strongly associated with metabolic traits, including body mass index, fasting glucose levels, type 2 diabetes, body fat content and feeding behavior. These findings underscore the translational value of MAP4K7-deficient fly and mouse models. Particularly, MAP4K7 appears to play a critical role in the metabolic conversion of dietary sugars into lipids, as levels of fatty acids and expression of lipogenesis genes such as *Fas* and *Acc* are markedly downregulated in sugar-fed msn RNA interference animals^91^.

## Conclusion

In this Review, we summarized the roles of MAP4K family kinases, comprising seven members involved in vital cellular processes. We outlined current insights into how these kinases operate within diverse intracellular signaling networks and their connections to cancer and other diseases.

Although numerous studies have underscored the anticancer potential of the MAP4K family or individual MAP4K kinases, significant gaps remain that hinder the translation of these findings into clinical applications. In particular, the development of MAP4K-specific inhibitors and microRNA (miRNA)-based therapeutics remains largely unexplored. This scarcity of targeted approaches represents a critical shortfall in the current research landscape, as few studies have focused on identifying or optimizing small-molecule inhibitors or miRNA strategies that selectively regulate MAP4K activity.

The lack of well-characterized inhibitors for each MAP4K kinase limits the ability to delineate their specific functional roles in cancer progression and constrains therapeutic advancement. Moreover, although miRNAs offer a promising mechanism for the posttranscriptional regulation of MAP4K expression, research into this regulatory axis is still insufficiently developed.

Here, we discussed the dual roles of MAP4K family kinases as both oncogenes and tumor suppressors in cancer. For example, MAP4K4 has been shown to function as an oncogene in several cancer types, including colorectal and pancreatic cancers, where it promotes cell proliferation, migration and metastasis. However, emerging evidence suggests that MAP4K4 may also act as a tumor suppressor in PDAC, where its depletion leads to enhanced tumor progression via hyperactivation of the AKT, ERK and mTORC signaling pathways. These findings indicate that MAP4K4’s function is highly context-dependent, influenced by the cellular environment and the interplay of multiple signaling networks. Current research suggests that MAP4K6 primarily acts as a tumor suppressor, playing a key role in regulating RAS-oncogene-induced growth arrest. Through the activation of the p38 MAPK signaling pathway, MAP4K6 induces cell cycle arrest and senescence-like phenotypes, thereby counteracting oncogenic transformation. Whereas MAP4K6’s involvement in stress-response signaling supports its tumor-suppressive potential, its possible oncogenic functions remain largely unexplored. Further investigation is needed to determine whether MAP4K6 may exhibit context-dependent oncogenic activity under specific genetic or environmental conditions that modify its signaling behavior.

These findings present conflicting evidence regarding the roles of specific MAP4K kinases across various cancer types, underscoring their context-dependent functions. Understanding these opposing roles is key to unraveling the complexity of MAP4K signaling and recognizing how a single kinase can contribute to both oncogenesis and tumor suppression under different circumstances. This broader perspective emphasizes the importance of context-specific strategies when targeting MAP4K family kinases in cancer therapy.

The development of MAP4K-specific inhibitors and miRNA-based interventions could not only help validate the therapeutic potential of these kinases but also pave the way for novel, more effective cancer treatment strategies. Therefore, future research should prioritize the discovery and optimization of selective MAP4K inhibitors and miRNA therapeutics, potentially advancing the field of precision oncology.
